# Povidone-iodine solution as SARS-CoV-2 prophylaxis for procedures of the upper aerodigestive tract a theoretical framework

**DOI:** 10.1186/s40463-020-00474-x

**Published:** 2020-10-27

**Authors:** Syed H. S. Naqvi, Martin J. Citardi, Davide Cattano, Luis Ostrosky-Zeichner, Mark I. Knackstedt, Ron J. Karni

**Affiliations:** 1 Department of Otorhinolaryngology-Head and Neck Surgery, McGovern Medical School, The University of Texas Health Science Center at Houston, 6431 Fannin Street, MSB 5.036, Houston, TX 77030 USA; 2 Department of Anesthesiology, McGovern Medical School, The University of Texas Health Science Center at Houston, Houston, TX 77030 USA; 3 Division of Infectious Diseases, Department of Medicine, McGovern Medical School, The University of Texas Health Science Center at Houston, Houston, TX 77030 USA

**Keywords:** Povidone-iodine, Betadine, SARS-CoV-2, Prophylaxis, COVID-19, Upper Aerodigestive tract

## Abstract

**Background:**

The COVID-19 pandemic has raised concerns of inadvertent SARS-CoV-2 transmission to healthcare workers during routine procedures of the aerodigestive tract in asymptomatic COVID-19 patients. Current efforts to mitigate this risk focus on Personal Protective Equipment, including high-efficiency filtration as well as other measures.

Because the reservoir for SARS-CoV-2 shedding is in the nasopharynx and nasal and oral cavities, the application of viricidal agents to these surfaces may reduce virus burden. Numerous studies have confirmed that povidone-iodine inactivates many common respiratory viruses, including SARS-CoV-1. Povidone-iodine also has good profile for mucosal tolerance. Thus, we propose a prophylactic treatment protocol for the application of topical povidone-iodine to the upper aerodigestive tract.

**Conclusion:**

Such an approach represents a low-cost, low-morbidity measure that may reduce the risks associated with aerosol-generating procedures performed commonly in otorhinolaryngology operating rooms.

**Graphical abstract:**

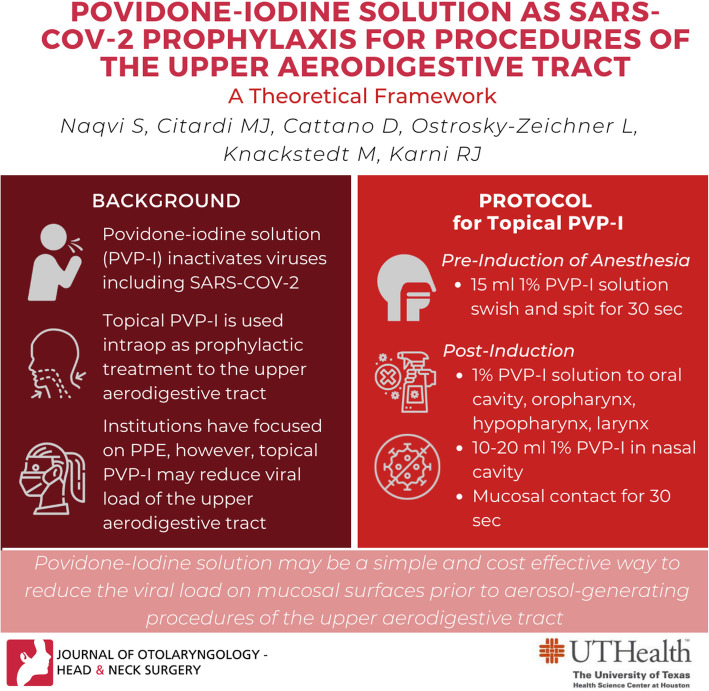

## Background

The COVID-19 pandemic has increased awareness of operating room transmission risks of the responsible virus, Severe Acute Respiratory Syndrome Coronavirus 2 (SARS-CoV-2). SARS-CoV-2 can remain aerosolized for at least three hours under experimental conditions and may persist for greater than 72 h on plastic and stainless steel surfaces; this creates substantial risks to all healthcare professionals [1]. The virus may reside in high concentrations in the nasal cavity, nasopharynx, oral cavity and oropharynx, and thus opportunities for dissemination of SARS-CoV-2 in the operating suite have been hypothesized [2, 3]. Intubation, as well as transoral and transnasal procedures, may pose a unique risk as viral particles may be aerosolized while performing these measures and contact with mucosa can be extensive. Various anecdotes confirm a relatively high prevalence of COVID-19 among otorhinolaryngologists, and for this reason, special precautions have been proposed for many aerosol-generating transnasal procedures [4]. Given that a minority of infected patients may remain asymptomatic, and that rapid and reliable screening remains limited, prevention against viral exposure has primarily focused on Personal Protective Equipment (PPE) [5–7]. Topical oronasal treatment with povidone-iodine (PVP-I), better known as Betadine™ (Avrio Health, LP), may be an effective method to immediately reduce the viral load of the upper aerodigestive tract and thus decrease the risk of inadvertent virus transmission.

Currently, there are no recommendation for routine anti-viral prophylaxis using PVP-I, despite a strong record of extensive viricidal activity. It is our contention that oronasal application of PVP-I may serve as a prophylaxis measure, alongside PPE, during invasive procedures involving the mucosa of the upper aerodigestive tract in the era of SARS-CoV-2 pandemic.

### Mechanism of action

PVP-I functions as an antiseptic through several mechanisms and is considered to have the broadest spectrum of action compared to other common antiseptics such as chlorhexidine [8, 9]. The two most potent antiseptic metabolites of PVP-I are molecular I_2_ and hypoiodous acid, which deliver free iodine. These free iodine molecules oxidize amino acids, nucleic acids and cell membranes [10]. Through oxidation of cell surface receptors, PVP-I prevents the attachment of viruses to cellular receptors [11].

### In vitro data

In 2006, Kawira et al. demonstrated inactivation of SARS-CoV-1 with various PVP-I dilutions from 0.23 to 1% at 2-min exposure time. In his discussion, Kawira writes: “PVP-I products for gargling and spraying the throat may have a prophylactic effect on SARS during outbreaks.” [12] In 2015, Eggers et al. reported that a 1% PVP-I gargle for 15 s reduced Middle East respiratory syndrome-related coronavirus (MERS-CoV) titer by greater than 99.99% [13]. In 2018, Eggers again demonstrated that both Severe Acute Respiratory Syndrome Coronavirus 1 (SARS-CoV-1) and MERS-CoV could be rapidly inactivated by PVP-I in concentrations as low as .23% applied for 15 s [14]. Other authors have reported similar viricidal effects on influenza, rotavirus, Ebola, HIV, adenovirus, polyomavirus, and hepatitis A [15–17]. Of note, PVP-I also has bactericidal effects against common oral pathogens such as *Klebsiella pneumonia* and *Streptococcus pneumonia* [9, 15, 18].

### In vivo studies

To our knowledge, there are no in vivo studies of oronasal application of PVP-I to reduce coronavirus infection. A Japanese randomized control trial of daily PVP-I gargle versus a control group showed an improvement to incidence rate of first upper respiratory tract infection [19]. A subsequent cost-effectiveness study of daily prophylactic PVP-I gargling suggests that this is an acceptable strategy when looking at quality of life days and cost [20].

Nagatake et al. reported a 50 % reduction in *Pseudomonas aeruginosa*, *Staphylococcus aureus* and *Hemophilus influenza* in 23 adults with chronic respiratory conditions by PVP-I gargling four times daily [21]. Shiraishi et al. reported a significant decrease in absence rate due to common cold and influenza in a middle school where the use of PVP-I gargle was encouraged. In this same study, PVP-I gargle was associated with a mean reduction rate of bacterial count by 99.4% [22].

Ogata et al. compared bacterial levels in the oropharynx at intubation and at the tip of the endotracheal tube after extubating, among patients using PVP-I gargle or tap water gargle. The bacterial levels in the oropharynx at intubation were markedly lower in the PVP-I gargle group. Tap water gargle patients had higher levels of bacterial colonization of the endotracheal tube tip (26.1% of tap water gargle patients versus none in the PVP-I gargle group for level 3 or 4 bacterial colonization) [23].

### Safety

Oral PVP-I gargle formulations are currently available as over-the-counter medications in many countries, including Japan and Canada. Rare cases of aspiration pneumonitis as well as thyroid dysfunction have been reported as side effects to povidone-iodine [24–27]. Cases of anaphylaxis, contact dermatitis and edema after exposure have also been reported [28–30]. Ingestion in high concentrations or quantities may lead to acute kidney injury and/or liver toxicity [31, 32]. PVP-I in low concentrations has not been known to stain teeth [33]. Topical oro-nasal PVP-I prophylaxis should not be considered in patients with iodine allergy or those undergoing radioiodine treatment. In a study of PVP-I oral gargle for cancer-associated oral mucositis, no mucosal irritation was reported [30, 34]. Of note, studies have shown that PVP-I is ciliotoxic in concentrations of 5% and 10% [35, 36].

## Proposed prophylactic treatment

Prior to the induction of anesthesia, each patient self-administers PVP-I as follows:

- 15 ml 1% PVP-I as a swish and spit for 30 s

The protocol recommendation above could potentially also be applied in a clinic setting, where aerosol generating procedures are frequently performed.

After general anesthesia has been induced,

- 1% PVP-I solution is applied to oral cavity, oropharynx, hypopharynx and laryngopharynx surfaces (for all transoral procedures or other procedures that cross these mucosal surfaces)
- 10–20 ml 1% PVP-solution is placed into the nasal cavity (for any transnasal procedure).
- Mucosal contact of the PVP-I solution is maintained for 30 s.

The rationale for using 1% PVP-I solution and 30 s mucosal contact time was determined on the basis of widespread over the counter availability and recommended instructions on usage of 1% PVP-I gargle and mouth wash in countries around the world [22].

## Conclusion

PVP-I has been used for more than 60 years as a topical antiseptic agent. Of note, PVP-I is viricidal against a wide range of viruses, including coronaviruses. Numerous reports confirm that low doses of PVP-I applied for short periods of time are extremely effective at reducing viral load. The safety profile of topical application of PVP-I to oral mucosa has been demonstrated. As health care settings develop new protocols around SARS-CoV-2 prophylaxis, the application of PVP-I solutions to the upper aerodigestive tract appears to be a low-cost and simple intervention for reducing viral burden from relevant mucosal surfaces.

Mucosal PVP-I application, deployed alongside existing protocols for PPE, may decrease the risk of contagion to healthcare personnel, especially in procedures which traverse mucosal membranes of the head and neck. Additional studies to examine the quantitative effect of PVP-I on viral load, the duration of effect, and the safety of oronasal application of PVP-I are warranted. Strong consideration for institution of this protocol should be given in light of the risks of inadvertent SARS-CoV-2 transmission during aerosol-generating procedures of the upper aerodigestive tract.

## Data Availability

Not applicable. Corresponding author available to answer unresolved questions upon request.

## Abbreviations

SARS-CoV-2: Severe Acute Respiratory Syndrome Coronavirus 2
PPE: Personal Protective Equipment
PVP-I: Povidone-iodine
